# Simple Protein Analysis by Droplet Paper Spray Ionization Mass Spectrometry with Polyolefin Silica-Based Paper

**DOI:** 10.3390/molecules28217339

**Published:** 2023-10-30

**Authors:** Sung Jun An, Youngjoo Kal, Minjoo Jeong, Sumin Kang, Byeongho Kwak, Hyunsoo Kim, Shihyeon Ryu, Sangwon Cha

**Affiliations:** Department of Chemistry, Dongguk University, Seoul 04620, Republic of Korea

**Keywords:** paper spray ionization, Teslin paper, protein, mass spectrometry, in situ analysis

## Abstract

Paper spray ionization mass spectrometry (PSI MS) has emerged as a notable method for the rapid analysis of biological samples. However, the typical cellulose-based paper tip is incompatible with protein detection due to the strong interaction between cellulose hydroxyl groups and proteins. In this study, we utilized a commercially available polyolefin-based synthetic paper, Teslin^®^, as an alternative PSI substrate for simple protein analysis. We have named this method “droplet PSI” MS, as the aqueous protein solution droplet retains its shape on the Teslin^®^ paper tip. For droplet PSI, no further chemical pretreatment was necessary for the Teslin^®^ substrate; the only required preparation was shaping the Teslin^®^ paper into a triangular tip. In droplet PSI MS, protein ion signals were instantly detected from a protein solution droplet upon applying a spray solvent in situ along with high voltage (HV). When compared with conventional PSI MS, our method demonstrated superior sensitivity. The droplet PSI MS utilizing Teslin^®^ also showcased flexibility in real-time observation of protein alterations induced by an acid additive. Additionally, the effects of spray solvent composition and the application method were discussed.

## 1. Introduction

Paper spray ionization (PSI) is one of the ambient desorption ionization methods for mass spectrometry (MS), in which analytes are extracted from a sample spot on a triangular paper tip by a solvent and transported via capillary action and/or bulk solution movement to the edge of the paper tip, where they are ionized in a manner like electrospray ionization (ESI) by a high voltage (HV) applied to the paper tip [1,2,3,4]. PSI MS has emerged as a simple and fast way to analyze drugs and metabolites directly from biological samples such as urine [5], blood [4], and tissue [6] with minimal or no sample preparation partly due to the filtering properties of the paper itself.

Conventional PSI MS, employing a filter or chromatography paper with a water (H_2_O)/methanol (MeOH) solvent, has been predominantly used for analyzing small molecules within a given sample. However, it has also found utility in the detection of proteins [1]. Moreover, noncovalent protein complexes in human blood residue, after the removal of plasma, were observed by PSI MS [7]. However, a significant challenge arises when attempting to detect proteins on a cellulose-based paper due to their strong interactions with the numerous hydroxyl groups present in the paper [4,8]. This interaction hinders their migration and desorption from the paper substrate, resulting in poor sensitivity in protein detection. In addition, when operating PSI in the dumping mode, wherein tens of microliters of solvent are applied to the paper tip at once, effective desolvation to facilitate protein ion detection may be difficult until the solvent is nearly depleted [4].

Several approaches have been attempted to address these limitations of PSI MS in protein detection. The simplest approach involved pre-wetting the paper tip with spray solvent prior to the protein sample loading [9]. This process could reduce the direct interaction between cellulose hydroxyl groups and proteins and improve protein transport through the resulting liquid film on the paper tip [9]. The second approach involves modifying the paper tip’s surface to reduce its hydrophilic nature. For example, silanization of cellulose hydroxyl groups was used to reduce the surface energy of the paper and increase its hydrophobicity [4,8]. When an aqueous-based protein solution was applied to the appropriately prepared hydrophobic paper, it could retain its form as a spherical droplet without wetting the paper [8]. Upon application of HV, electrostatic charging induced the emission of charged microdroplets from a spherical droplet on a hydrophobic paper [8]. In this process, the unfolding of proteins could be further promoted, resulting in a higher charge state distribution (CSD) of a given protein compared to conventional PSI or nanoESI [8]. In addition to silanization, the impregnation of polystyrene onto the paper surface was also used to the increase hydrophobicity of a paper tip to facilitate the sensitive detection of proteins [10].

The third approach is also the surface modification of the paper tip to increase the desorption and ionization efficiency of proteins. The paper coated with basic NH_2_-bonded silica microspheres showed a significant 10- to 170-fold improvement in signal-to-noise ratio (*S*/*N*) compared to untreated filter paper when analyzing lysozyme and myoglobin [9]. By coating the paper tip with carbon nanotubes (CNTs) to increase its surface area and conductivity, the in-gel protein could be extracted and ionized directly from the paper tip [11]. Furthermore, a study assessing the adsorption and desorption capabilities of Zr-based metal–organic frameworks using PSI MS revealed that paper tips coated with UiO-66(Zr) demonstrated improved desorption and ionization of myoglobin [12].

An alternative approach to enhance protein sensitivity involves utilizing the PSI platform but with a spraying tip composed of different materials instead of cellulose-based paper. For instance, when a dialysis membrane spraying tip is positioned on top of a filter paper and biological fluid is applied, small molecules excluding proteins permeate the filter paper [13]. This cleanup process enables the detection of proteins remaining on the membrane with high sensitivity. Moreover, a porous polyethylene tip treated with CNTs exhibited detection limits for protein detection that were 10–100 times lower than CNT-treated paper [14]. In another case, a gold wire for electrical contact was placed on a triangular Teflon^TM^ tip and was utilized for protein detection [15].

In this study, we developed a PSI MS platform for immediate in situ analysis of protein solutions using a commercially available, polyolefin silica-based substrate, known as Teslin^®^, a synthetic paper widely used as waterproof printing material. PSI MS using Teslin^®^ paper has previously been employed for achieving high sensitivity in drug analysis and monitoring lipid metabolite alterations induced by COVID-19 infection [16,17]. An aqueous-based protein sample loaded on Teslin^®^ paper remains as a droplet as on other hydrophobic substrates [8,15]. We have optimized PSI parameters to enable the immediate detection of protein ion signals directly from this droplet, eliminating the need for air-drying procedures.

## 2. Results and Discussion

### 2.1. Set up of Droplet PSI MS for Protein Detection

**Figure 1:**
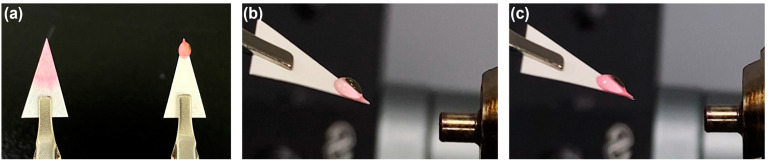
(**a**) A photo showing (**left**) a filter paper tip and (**right**) a Teslin^®^ paper tip after loading a protein solution in 50 vol% aqueous MeOH. Rhodamine 6G was added to the solution for better visualization (**b**,**c**). Photos of droplet PSI of a protein solution in 50% aqueous MeOH (**b**) before and (**c**) after applying HV. Refer to Supplementary Video S1 to see the droplet PSI in action.

The Teslin^®^ substrate is composed of a hydrophobic high molecular weight polyolefin matrix and a hydrophilic filler, primarily silica. It is known to be resistant to alcohols, esters, weak acids, and some strong acids, including hydrochloric acid and sulfuric acid. Due to its hybrid nature (being both hydrophobic and hydrophilic), it is possible that a droplet can maintain its shape while exhibiting a low contact angle on its surface. Figure 1a shows how an aqueous MeOH droplet retains its shape on a Teslin^®^ paper tip, as compared to a cellulose-based filter paper tip. When aqueous MeOH solutions were applied to a given Teslin^®^ substrate in 10 vol% increments of MeOH content, the droplets retained their shape up to a concentration of 60 vol% MeOH. However, at 70 vol% MeOH, they lost their shape and spread on the substrate surface. The surface tension values of 60/40 MeOH/H_2_O and 70/30 MeOH/H_2_O at 25 °C are known to be in the ranges of 29.8–32.9 mN/m and 27.5–29.8 mN/m, respectively [18,19]. Therefore, the critical surface energy of a given Teslin^®^ substrate was roughly estimated to be in the range of 27.5–29.8 mN/m.

As shown in Figure 1b, 4 pmol/μL of cytochrome C in aqueous MeOH was applied to the Teslin^®^ substrate, and the conditions under which PSI could occur were tested. Initially, we varied the content of MeOH and found that spray ionization did not occur from droplets containing 30 vol% or less MeOH, even when voltages up to +3.7 kV were applied; moreover, the droplet itself was sucked into the mass spectrometer inlet when higher HVs were applied. When voltages ranging from +3.5 kV to +4 kV were applied to protein droplets containing 35 to 60 vol% MeOH, spray action was observed, as depicted in Figure 1c. Comparing Figure 1c to Figure 1b, the droplet was slightly compressed, and the solution was stretched towards the tip, forming a Taylor cone-like shape at the tip. The changes in the droplet and the spraying process after applying HV can be seen more clearly in Supplementary Video S1. The spraying and ionization processes in droplet PSI with Teslin^®^ paper are expected to resemble those in PSI with silanized hydrophobic paper [8]. Upon HV application, charged microdroplets are emitted from the droplet when the pressure or repulsive force generated by the accumulated electrostatic charges at the droplet–air interface surpasses the pressure difference between the interior and exterior of the droplet [8].

**Figure 2:**
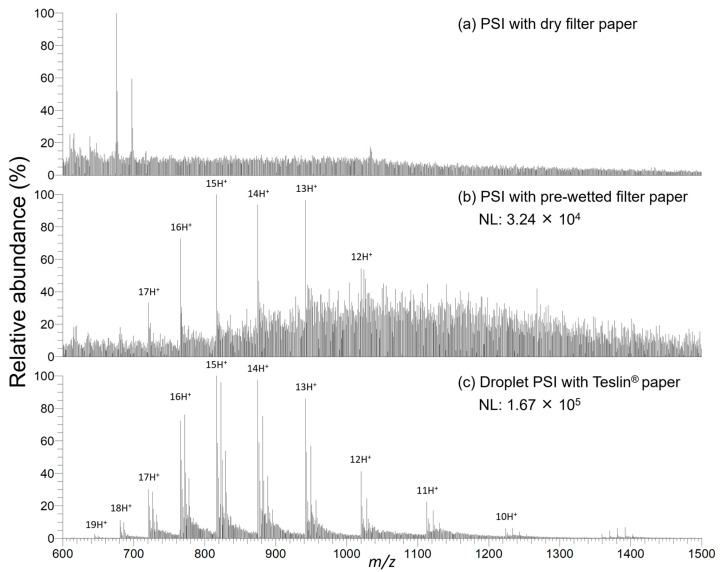
PSI mass spectra of cytochrome C in 50 mM AmAc solution under various PSI conditions: (**a**) 1 μL of 4 pmol/μL protein solution was applied to a filter paper tip, left to dry, followed by the application of 10 μL of spray solvent and HV; (**b**) 1 μL of 4 pmol/μL protein solution was loaded onto a pre-wetted filter paper tip with spray solvent. The spray solvent for (**a**,**b**) was 1% (*v*/*v*) FA in 50/50 MeOH/H_2_O. (**c**) 2.5 μL of 1.6 pmol/μL protein solution was first spotted onto the Teslin^®^ paper tip, followed by the addition of 2.5 μL of spray solvent to the protein droplet formed on the Teslin^®^ paper tip, after which HV was applied. The spray solvent for (**c**) was 1% (*v*/*v*) FA in MeOH. Note that the amount of protein loaded in (**a**–**c**) was the same (4 pmol). NL denotes the normalized level.

We utilized a 50 vol% aqueous MeOH droplet containing 1% (*v*/*v*) formic acid (FA) as the default condition, considering the stability of the droplet during droplet PSI, and the intensity and stability of the protein ion signals. In other words, when the MeOH content was in the range of 35–45 vol%, the PSI phenomenon could be observed, but the spray was often disrupted, or a low *S*/*N* was observed for the protein ion signals. PSI mass spectra of cytochrome C in 50 mM ammonium acetate (AmAc) obtained using either a cellulose-based filter paper tip or a Teslin paper tip are shown in Figure 2. No protein signal could be observed in the conventional PSI process, where the protein solution is spotted, and then dried, followed by the application of HV and the spray solvent (Figure 2a). In contrast, in the modified PSI process [9], where the protein solution is loaded onto a filter paper tip pre-wetted with the spray solvent to reduce direct interaction between cellulose hydroxyl groups and proteins, noisy but discernible protein ion signals were detected (Figure 2b). In marked contrast to the conventional PSI results, the droplet PSI MS with a Teslin^®^ paper tip yielded a spectrum in which the CSD of cytochrome C was clearly observed (Figure 2c). For cytochrome C and myoglobin, droplet PSI MS showed at least a 20-fold *S*/*N* improvement over conventional PSI. These results suggest that droplet PSI MS employing a Teslin^®^ paper tip exhibits superior sensitivity compared to conventional PSI MS using a cellulose filter paper.

### 2.2. Procedure of Droplet PSI MS

**Figure 3:**
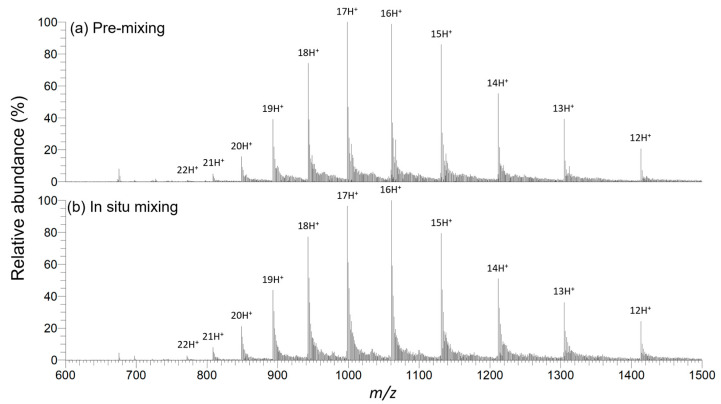
Droplet PSI mass spectra of 6 pmol/μL myoglobin in 50 mM AmAc with different spray solvent application methods. (**a**) A 1:1 mixture of protein solution and 1% (*v*/*v*) FA in MeOH was prepared 10 min before analysis and 5 μL of this mixture was applied to a Teslin^®^ paper tip; (**b**) 2.5 μL of protein solution was first spotted onto the Teslin^®^ paper tip and 2.5 μL of 1% (*v*/*v*) FA in MeOH was added to this protein solution droplet in situ right before analysis.

Next, a comparison was made between two procedures for droplet PSI: pre-mixing the protein solution with the spray solvent and loading the resulting mixture onto a Teslin^®^ paper tip versus in situ mixing of the spray solvent with the protein solution already loaded onto the Teslin^®^ paper tip. Droplet PSI mass spectra of 6 pmol/μL myoglobin in 50 mM AmAc obtained by these two procedures are shown in Figure 3. The following are the subtle differences between these two spectra: First, although the difference between the 16+ and 17+ ion intensities in each spectrum was slight, the most abundant charge state (MACS) in pre-mixing was 17+ (Figure 3a), while the MACS in in situ mixing was 16+ (Figure 3b). Second, *S*/*N* for the ion (M + 16H)^16+^ at *m*/*z* 1060 was slightly lower in the case of in situ mixing (*S*/*N* = 33) compared to pre-mixing (*S*/*N* = 36). These differences are reasonable given the proteins were exposed to a denaturing environment for a longer period under pre-mixing conditions compared to in situ mixing.

Despite these differences, the two spectra are very similar in terms of CSDs and the average charges of the observed CSDs (Z_AVE_ = 16.1 for pre-mixing and 16.3 for in situ mixing) [20]. One possible reason for the similarity in results, despite the shorter duration of in situ mixing, is the droplet vibration that occurs during the spraying process. This droplet vibration, observed in hydrophobic PSI MS with silanized paper [8] and also seen in this study (Supplementary Video S1), is believed to facilitate quicker mixing when the spray solvent is applied to the protein solution droplet in situ. Overall, we believe that analyzing proteins by in situ mixing of spray solvent offers unique advantages given the procedural simplicity and flexibility of the method. The flexibility of the in situ mixing method is demonstrated in the next section, which covers the in situ addition of an acid additive during droplet PSI MS.

### 2.3. In Situ Addition of an Acid Additive during Droplet PSI MS

**Figure 4:**
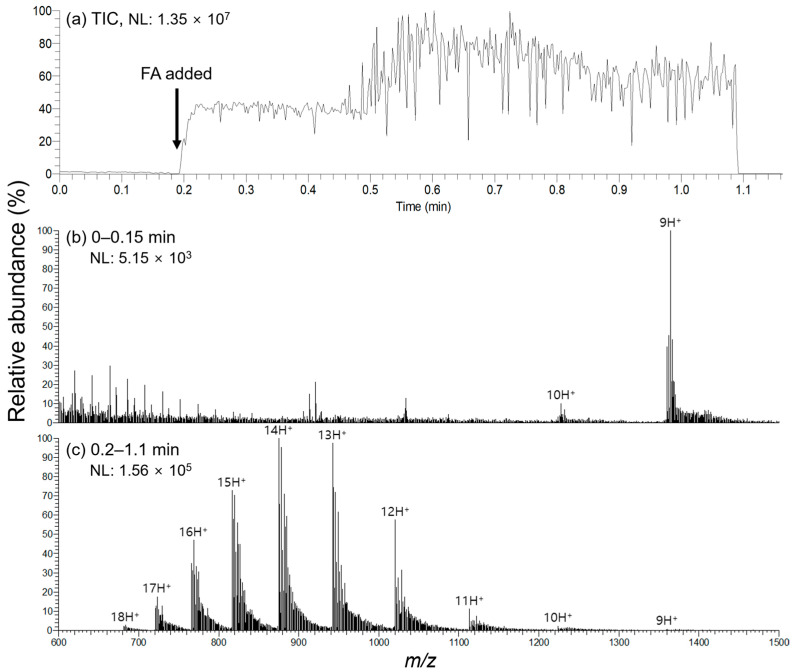
Addition of an acid additive during droplet PSI MS: Initially, 2.5 μL of 4 pmol/μL cytochrome C in 50 mM AmAc was spotted onto the Teslin^®^ paper tip. This was followed by the addition of 2.5 μL of MeOH just before performing analysis. Subsequently, HV was applied, and the mass spectrum was collected. Around 0.2 min after initiating data collection, 2.5 μL of 1% (*v*/*v*) FA in MeOH was added to the droplet without turning off HV. Displayed are (**a**) the total ion chronogram (TIC), and averaged mass spectra collected during (**b**) 0–0.15 min and (**c**) 0.2–1.1 min. NL denotes the normalized level.

We conducted an experiment wherein an acid additive was introduced during the spraying process to determine whether the acid-induced protein denaturation could be observed in real time using droplet PSI MS. The total ion chronogram (TIC) and time-segmented mass spectra collected during the sequential addition of MeOH and 1% (*v*/*v*) FA in MeOH to cytochrome C in 50 mM AmAc on the droplet PSI MS platform are depicted in Figure 4. Table 1 lists CSD information for cytochrome C ions before and after the addition of FA. In addition, a video for the above analysis process is presented in Supplementary Video S2. As seen from the TIC, the ion intensity markedly increases with the addition of FA near 0.2 min (Figure 4a), and a distinct change in CSD before and after FA addition is illustrated in Figure 4b,c and Table 1. The phenomenon of CSD shifting to a higher charge as pH decreases has been well-documented [20,21]. However, the notable point here is that the open environment of droplet PSI MS can provide the flexibility to easily observe such trends in real time from a single sample. In other words, this suggests that although the presence of organic solvents impedes the observation of proteins in their native state, droplet PSI MS can be employed to efficiently investigate alterations in proteins induced by various factors, including pH, salt concentration, or solvent composition, as previously demonstrated with desorption ESI (DESI) [22,23].

On the other hand, it should be noted that the CSD shift to a slightly lower charge was observed in the spectrum depicted in Figure 4c compared to that of Figure 2c. There are possible contributing factors to this phenomenon. The experiment for Figure 4c had twice the added amount of MeOH as that for Figure 2c while keeping the aqueous protein solution volume constant. Therefore, the droplet in Figure 2c has lower MeOH content and thus higher surface tension compared to the droplet in Figure 4c. It was hypothesized, and supported by many studies, that a droplet with higher surface tension can hold more charges before reaching the Rayleigh limit [20,24]; our data were also consistent with this hypothesis. In addition, the more diluted concentration of FA in Figure 4c could also serve as another contributing factor to this phenomenon.

### 2.4. Effect of Solvent Composition in Droplet PSI MS

All the droplet PSI MS experiments described above were performed using MeOH. In addition, droplet PSI MS was also performed using other ESI-compatible organic solvents, aprotic acetonitrile (ACN), and protic isopropyl alcohol (IPA), and the results were compared to those using MeOH. The surface tension values (γ in mN/m) of pure H_2_O, MeOH, ACN, and IPA are 71.99, 22.07, 28.66, and 20.93 mN/m, respectively [20]. Based on these values alone, one would expect that a larger amount of organic solvent needs to be contained in the droplet for droplet PSI to occur with ACN than with MeOH. However, the droplet with 50 vol% ACN spread more than that with 50 vol% MeOH on a Teslin^®^ paper tip. In fact, for a binary mixture of organic solvent and water in a 1:1 ratio, the surface tension value of aqueous ACN (γ~32.9 mN/m) is lower than that of aqueous MeOH (γ~35.4 mN/m) [18,25,26]. This may be attributed to the endothermic process of mixing aprotic ACN with water, which breaks the hydrogen bond network of water molecules [27]. We found that the droplet containing 20–35% ACN was suitable for droplet PSI MS analysis of proteins. In the case of IPA, as expected from its surface tension value, less than 20 vol% IPA in the droplet was appropriate for the droplet PSI process.

**Figure 5:**
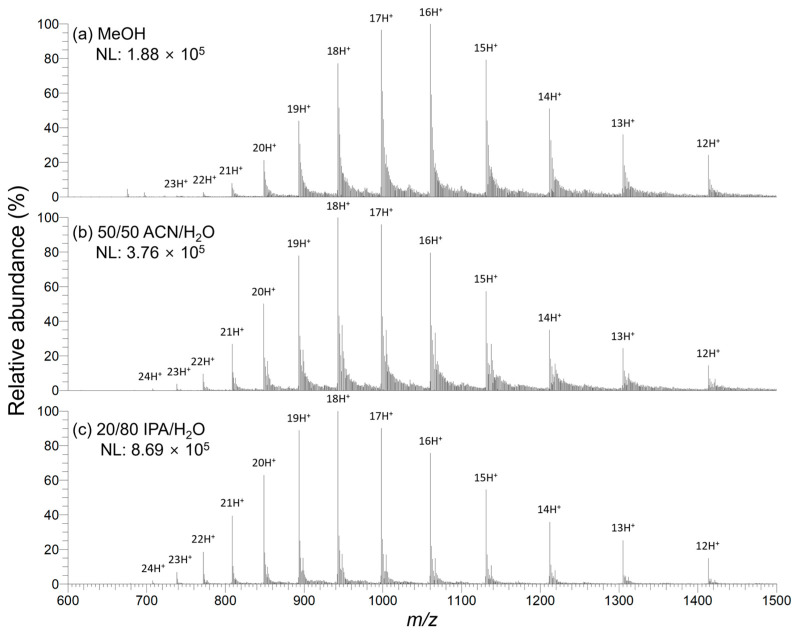
Droplet PSI mass spectra of 6 pmol/μL myoglobin in 50 mM AmAc with various spray solvent compositions, 1% (*v*/*v*) FA in (**a**) MeOH, (**b**) 50/50 ACN/H_2_O, and (**c**) 20/80 IPA/H_2_O. Initially, 2.5 μL of protein solution was spotted on the Teslin^®^ paper tip. Then, 2.5 μL of spray solvent was added to the protein droplet formed on the Teslin^®^ paper tip, followed by the application of HV. Solvent composition in each figure panel indicates the spray solvent composition. After mixing with the protein solution, the final organic solvent content in the droplet will be half of the indicated content. NL denotes the normalized level.

Figure 5 displays the droplet PSI mass spectra of myoglobin measured at the optimized organic solvent-to-water ratio for each organic solvent, and Table 2 lists the CSD information for myoglobin ions alongside the *S*/*N* for the 16+ ion at *m*/*z* 1060 in each solvent condition. ACN- or IPA-based spray solvent exhibited the shift of the CSD to a higher charge (lower *m*/*z*) compared to MeOH-based spray solvent. This is likely due to the increased surface tension of the droplets as the water content increases, which contributes to a higher charge, as mentioned earlier [20,24]. Disregarding the FA, AmAc, and myoglobin that were present in equal amounts in each droplet, the surface tension values of the droplets were estimated to be γ ≈ 35.4 mN/m for 50/50 MeOH/H_2_O, γ ≈ 38–39 mN/m for 25/75 ACN/H_2_O, and γ ≈ 41.2 mN/m for 10/90 IPA/H_2_O, based on the surface tension values reported in the literature [18,19,25,27].

Interestingly, *S*/*N* for the 16+ ion at *m*/*z* 1060 was significantly higher for the IPA-based spray solvent compared to other spray solvents. This observation contrasts with the general ESI concept, which posits that a higher proportion of organic solvent results in finer charged droplets, thereby promoting desolvation and improving ionization efficiency [28]. The reason for this discrepancy remains unclear. However, it is speculated that higher water content further reduces the interaction between the droplet and the Teslin^®^ substrate. This reduction may lead to a greater accumulation of electrostatic charges at the air–droplet interface, facilitating the generation of charged microdroplets.

Lastly, we also measured myoglobin in AmAc solution with droplet PSI MS by varying the concentration of FA or by using acetic acid (AA) as an acid additive instead of FA. It is important to note that equal volumes of spray solvent and sample were mixed to form the droplet, leading to the acid additive’s concentration being halved in the final droplet. For protein samples dissolved in AmAc solution, reproducible signals were achieved with acid additive concentrations of 1% (*v*/*v*) or more. In contrast, for protein samples dissolved in pure water, signals were attainable using a spray solvent containing either 0.1% (*v*/*v*) FA or 0.5% (*v*/*v*) AA. As the concentration of FA in the spray solvent varied at 1%, 2%, and 10% (*v*/*v*), the Z_AVE_ values of myoglobin CSD correspondingly showed an increase, with values of 15.9, 16.0, and 17.2, respectively. However, we did not find significant differences in *S/N* or overall CSD shape. AA resulted in an overall lower Z_AVE_ than FA; the Z_AVE_ values were 15.2, 15.7, and 15.9 as AA concentration increased to 1%, 2%, and 10% (*v*/*v*), respectively. However, when considering the molecular weight and density values of FA and AA (46.03 g/mol and 1.22 g/mL for FA and 60.05 g/mol and 1.05 g/mL for AA), FA has approximately 1.5 times higher molarity than AA at the same volume percentage (*v*/*v*). Therefore, the difference in Z_AVE_ between FA and AA may be due to the concentration difference rather than the properties of each acid.

## 3. Materials and Methods

### 3.1. Materials

Cytochrome C and myoglobin were purchased from Sigma-Aldrich Chemical Co. (St. Louis, MO, USA) and used without further purification. AmAc and organic solvents, including MeOH, IPA, and ACN were also obtained from Sigma-Aldrich Chemical Co. (St. Louis, MO, USA). Acid additives, FA and AA, were purchased from Tokyo Chemical Industry (Tokyo, Japan). Polyolefin silica-based synthetic paper, Teslin^®^ SP700 paper (0.178 mm in thickness), was obtained from PPG Teslin^®^ Substrate Products (Monroeville, PA, USA). Cellulose-based paper used for conventional PSI MS was Whatman grade 6 filter paper (0.180 mm in thickness, Whatman International Ltd., Maidstone, UK).

Protein stock solutions (1 mg/mL) were prepared in 50 mM AmAc and diluted with the same solution for both conventional and droplet PSI MS analyses.

### 3.2. PSI MS

The PSI MS analysis was performed by linking a custom-built PSI probe to the integrated HV supply of a linear ion trap mass spectrometer (Thermo Finnigan LTQ XL, Mountain View, CA, USA). A triangular paper tip (2 mm base and 5 mm height) was held at an angle of approximately 30 degrees at 5–7 mm from the mass spectrometer inlet by a clip connected to the HV supply.

In conventional PSI MS utilizing filter paper, two measurement methods were employed: (1) One microliter of protein solution was spotted onto the paper, left to dry, and then ten microliters of 1% (*v*/*v*) FA in 50/50 MeOH/H_2_O was applied while HV was applied. (2) The paper tip was first moistened with 1% (*v*/*v*) FA in 50/50 MeOH/H_2_O. Subsequently, 1 μL of protein solution was loaded onto it before applying HV [9].

Droplet PSI MS with Teslin^®^ paper was also conducted in two ways: (1) The protein solution and spray solvent were pre-mixed in a 1:1 volume ratio. Next, 5 μL of this mixture was placed on the front of the Teslin^®^ paper tip, followed by HV application. (2) Initially, 2.5 μL of the protein solution was added to the Teslin^®^ paper. Then, 2.5 μL of spray solvent was added to the droplet and mixed in situ, and then HV was applied. The water-to-organic solvent ratio in the spray solvent varied between 80:20 and 0:100 (H_2_O: organic solvent). Concentration of an acid additive varied from 1 to 10% (*v*/*v*) in the spray solvent. For more detailed spray solvent conditions and experimental procedures, please refer to the Results and Discussion section.

Mass spectra were collected in the positive ion full scan mode and spray voltage and tube lens voltage were set to +3.5–4.5 kV and 80 V, respectively. Temperature and voltage for the ion transfer capillary were set to 200 °C and 35 V, respectively.

## 4. Conclusions

In this study, we developed an alternative PSI MS platform, droplet PSI MS, with polyolefin silica-based synthetic paper, Teslin^®^, for simple and sensitive analysis of proteins. After careful optimization in droplet solution composition with ESI-compatible organic solvents, the protein solution could maintain its shape on a Teslin^®^ substrate, and charged microdroplets containing protein ions could be emitted directly from this droplet upon HV application. Based on the results of this study, it is believed that the key parameter for the droplet PSI process is the surface tension of the droplet. Compared to conventional cellulose-based PSI, Teslin^®^-based droplet PSI not only exhibited a significant increase in sensitivity but also facilitated more immediate protein signal acquisition by eliminating the sample drying process. Moreover, the open environment of droplet PSI facilitates the introduction of a reagent, such as a weak acid, to a protein solution droplet maintained on a Teslin^®^ substrate in the middle of the analysis. This allows for real-time observation of protein ion signal changes in the denaturing environment induced by the additive. Overall, based on its procedural simplicity and flexibility, we demonstrated the potential of droplet PSI MS for straightforward and immediate protein analysis.

## Figures and Tables

**Table 1:** CSD information of cytochrome C observed before and after adding FA during droplet PSI MS data acquisition (Figure 4b,c).

Condition	Z_HOCS_^1^	Z_MACS_^2^	Z_AVE_^3^
Before adding FA	10	9	9.1
After adding FA	18	14	13.9

^1^ Z_HOCS_ denotes the highest observed charge state. ^2^ Z_MACS_ denotes the most abundant charge state. ^3^ Z_AVE_ denotes the average charge of the observed CSDs. <Z> = ∑(z*I_z_*)/∑(*I_z_*), where z is the charge state and *I_z_* is the integrated abundance of each respective protein ion.

**Table 2:** CSDs of myoglobin and *S*/*N* values for the 16+ ion at *m*/*z* 1060 observed by droplet PSI MS with various spray solvents (Figure 5a–c).

Spray Solvent ^1^	Z_HOCS_	Z_MACS_	Z_AVE_	*S/N* for 16+
MeOH	23	16	15.9	33
50/50 ACN/H_2_O	24	18	17.0	32
20/80 IPA/H_2_O	24	18	17.9	103

^1^ All spray solvents contained 1% (*v*/*v*) FA, and all indicated ratios are in vol%.

## Data Availability

The data presented in this study are available on request from the corresponding author.

## Supplementary Materials

The following supporting information can be downloaded at: https://www.mdpi.com/article/10.3390/molecules28217339/s1, Video S1: Droplet paper spray in action upon HV application; Video S2: Procedure of droplet PSI MS analysis for in situ addition of an acid additive during spraying process (4× speed playback).
